# Potential for Early Fracture Risk Assessment in Patients with Metastatic Bone Disease Using Parametric Response Mapping of CT Images

**DOI:** 10.18383/j.tom.2015.00154

**Published:** 2015-12

**Authors:** Benjamin A. Hoff, Michael Toole, Corrie Yablon, Brian D. Ross, Gary D. Luker, Catherine Van Poznak, Craig J. Galbán

**Affiliations:** 1 Departments of Radiology and; 2 Internal Medicine, Center for Molecular Imaging, University of Michigan, Ann Arbor, MI

**Keywords:** computed tomography, parametric response map, vertebral compression fractures, bone metastases, skeletal-related event

## Abstract

Pathologic vertebral compression fractures (PVCFs) cause significant morbidity in patients with metastatic bone disease. Limitations in existing clinical biomarkers leave clinicians without reliable metrics for predicting PVCF, thus impeding efforts to prevent this severe complication. To establish the feasibility of a new method for defining the risk of a PVCF, we retrospectively analyzed serial computed tomography (CT) scans from 5 breast cancer patients using parametric response mapping (PRM) to quantify dynamic bone miniral density (BMD) changes that preceded an event. Vertebrae segmented from each scan were registered to the same spatial frame and voxel classification was accomplished using a predetermined threshold of change in Hounsfield units (HU), resulting in relative volumes of increased (PRM_HU+_), decreased (PRM_HU−_), or unchanged (PRM_HU0_) attenuation. A total of 7 PVCFs were compared to undiseased vertebrae in each patient serving as controls. A receiver operator curve (ROC) analysis identified optimal imaging times for group stratification. BMD changes were apparent by an elevated PRM_HU+_ as early as 1 year before fracture. ROC analysis showed poor performance of PRM_HU−_ in stratifying PVCFs versus controls. As early as 6 months before PVCF, PRM_HU+_ was significantly larger (12.9 ± 11.6%) than control vertebrae (2.3 ± 2.5%), with an area under the curve of 0.918 from an ROC analysis. Mean HU changes were also significant between PVCF (26.8 ± 26.9%) and control (−2.2 ± 22.0%) over the same period. A PRM analysis of BMD changes using standard CT imaging was sensitive for spatially resolving changes that preceded structural failure in these patients.

## Introduction

Bone metastases occur in approximately 70% of patients with metastatic breast cancer, and bone is the most common site of metastasis for patients with estrogen receptor-positive (ER+) disease (1). The spine is involved in approximately 20% of patients who have only a solitary metastatic bone lesion and in approximately 50% of patients with multiple bone lesions (2). Without bone-directed therapies, the estimated yearly incidence of skeletal-related events (SREs) is 3.5 (3), with a median incidence of 1.3 for pathologic vertebral compression fractures (PVCFs) (4). Breast cancer metastases to vertebral bodies present particularly devastating complications, including vertebral collapse with spinal cord compression (5–7). Metastases to vertebrae and other bones also produce pain, limited mobility, hypercalcemia, and poor quality of life. In particular, complications of spinal cord compression and hypercalcemia represent oncologic emergencies (8). Therapies for breast cancer metastases to vertebrae and other bones cost an estimated $4.2 billion in 1998 US dollars, emphasizing the tremendous burden to society and potential impact of new biomarkers for impending pathologic vertebral compression fractures (9).

Bisphosphonates such as zoledronic acid and the monoclonal antibody-targeting receptor activator of nuclear factor kappa-β ligand denosumab are the current bone-directed therapies that function to inhibit osteoclast activity. Bisphosphonates decrease the risk of SREs, including PVCFs, by approximately one-third, and denosumab may further improve the control of SREs by another 20% (10, 11). Although these state-of-the-art drugs decrease the risk of fractures, PVCFs and other SREs still occur frequently in these patients. In a phase III study comparing denosumab to zoledronic acid in women with metastatic breast cancer involving the bone at a median follow-up of 17 months, lumbar PVCFs were identified in 3.4% of patients on denosumab and 5.5% of patients receiving zoledronic acid (11). To optimize the use of these drugs to prevent complications of bone metastases, biomarkers are needed to identify individuals at an increased risk of PVCF. Moreover, predictive biomarkers for PVCFs would accelerate the development and effective implementation of new treatments for vertebral metastases, including external beam radiation therapy, radiopharmaceuticals, chemotherapy drugs, endocrine therapy, and kyphoplasty (12, 13).

Predicting individuals who are at the greatest risk for developing a PVCF would provide opportunities for earlier treatment and the design of clinical trials to prevent fractures. However, existing biomarkers for predicting pathologic fractures have limited efficacy. Biochemical markers such as serum alkaline phosphatase and urinary deoxypyridoline have shown some promise for predicting pathological fractures (14). Bone mineral density—measured by dual x-ray absorptiometry (DXA) or quantitative computed tomography (CT)—are used clinically to diagnose osteoporosis and related fracture risk (15). However, osteoporosis is not the only risk factor for fracture in cases of metastatic bone disease, which also causes localized areas of bone remodeling and resulting weakness. Current clinical methods for predicting bone fracture rely primarily on patient demographics and history as well as whole-bone measurements, which may not be sensitive to localized changes in bone mineral density (BMD) (15). As a result, new methods for predicting very early changes in bone mineralization that presage a future pathologic fracture are needed.

We previously developed the image analysis technique parametric response mapping (PRM) to quantify temporal changes in imaging data on a voxel-by-voxel basis (16–19). In a rat model of osteoporosis, we demonstrated that PRM of quantitative CT data sensitively detected spatially defined changes in BMD (20). Capitalizing on these preclinical data, we performed a retrospective clinical study to establish proof of concept for using PRM for detecting and quantifying early, spatially localized changes in BMD in breast cancer metastases to vertebrae prior to PVCF. Overall, this study defines PRM metrics for changes in BMD associated with PVCF risk in patients with metastatic breast cancer. Results of this study set the stage for future prospective studies establishing PRM of bone CT data as a novel imaging biomarker for PVCFs and other SREs.

## Methodology

### Patients

Retrospective imaging and clinical data were obtained from 5 patients at the University of Michigan and approved by an institutional review board. Patients were included in the study based on confirmed PVCF using CT and more than 3 scans pre-PVCF. An additional 2 cases of osteolytic lesions were found for anecdotal analysis despite the fact that fractures were not observed. All images were reviewed by a musculoskeletal radiologist to confirm the location and date of the fracture, to assess for other fractures, and to identify the vertebral body with the least amount of metastatic disease for use as a control. Clinical information was extracted from medical records, including patient characteristics (age, gender, ethnicity, date of birth, date of death), oncologic history (stage at diagnosis, ER/PR/Her2 status of primary and metastatic disease, and date of metastatic bone disease), history of oncologic treatment (including chemotherapy, hormonal therapy, and prior radiation therapy), bone-modifying agents or procedures (kyphoplasty and calcium, vitamin D, bisphosphonate, and steroid use), clinical characteristics proximal to compression fracture (back pain, indication for imaging), and treatment of vertebral fracture.

### PRM

PRM analysis was performed as previously described using software developed in MATLAB (MathWorks, Natick, MA) (20). Briefly, serial CT images, expressed in Hounsfield units (HU), were cropped around each individual vertebral body of interest, and bone volumes of interest were manually contoured on the baseline image to encompass the individual bone volume. All longitudinal images were registered to the first available image time point using Elastix open-source software (21). Registration was automatic and assumed rigid-body geometry, ie, rotation and translation. PRMs of quantitative CT as expressed in HU (PRM_HU_) were generated between time points by first calculating the difference between HU values for each voxel. Individual voxels were classified based on the extent of change observed in attenuation (ΔHU). Voxels that yielded a ΔHU greater than a receiver operating curve (ROC)-optimized threshold (ξ) were designated red (ΔHU > ξ), those that decreased by more than the threshold were designated blue (ΔHU≤-ξ), and those that did not significantly change from baseline were designated green. Because of a lack of true control imaging data, 100 HU was arbitrarily chosen for longitudinal PRM analysis. Volume fractions of the total bone volume were calculated for the 3 classifications: PRM_HU+_ (red voxels denoting increased HU), PRM_HU−_ (blue voxels denoting decreased HU), and PRM_HU0_ (green voxels denoting unchanged HU, graphed as 100% − PRM_HU0_ for easy comparison). For longitudinal tracking of PRM values, each time point was analyzed against the baseline image.

### Data and Statistical Analysis

**Figure 1.**
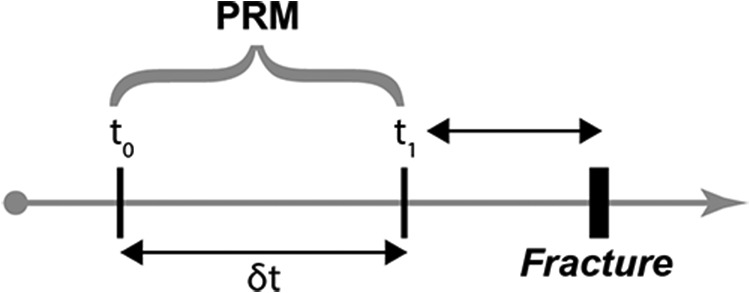
Schematic diagram showing analysis time intervals for generating PRM values from CT scans. Here *t*_1_ refers to the time between the second PRM and resulting fracture, and δ*t* refers to the time spacing between PRM values (*t*_1_ − *t*_0_).

To assess the ability of PRM measurements to stratify the PVCF cases versus their respective controls, 3 variables were explored: (1) the threshold that designated a significant change in HU within a voxel (ξ = ±50, 100, 150, or 200 HU); (2) the second time point relative to the date of fracture (*t*_1_ = 3, 6, 9, or 12 months; Figure 1); and (3) the time between serial images, acquired at *t*_0_ and *t*_1_, for PRM analysis (δ*t* = 3, 6, 9, or 12 months; Figure 1). An ROC analysis was used for evaluating the ability of PRM metrics to differentiate between fracture and control vertebrae. The area under the ROC curve (AUC_ROC_) was calculated as a summary statistic for selecting the optimal combination of PRM threshold and imaging time points, with a greater value indicating more accurate results. The cutoff value for discriminating between groups was then determined using the accuracy measure (ACC), defined as *ACC* = (*TP* + *TN*)/*n*, where *TP* is number of true positive results, *TN* is true negative, and *n* is the total number of samples. A higher value indicates a more accurate cutoff. Group comparisons (fracture versus control) were performed using a paired 2-tailed Student's *t* test, and significant difference between groups was defined as *P* < .05. All group statistics and plots are depicted as group mean with errors representing the SD.

## Results

### PRM for Predicting Fracture

**Figure 2.**
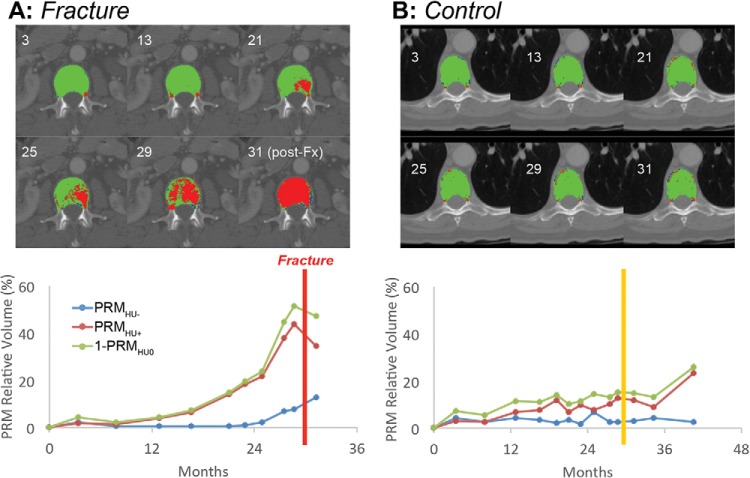
Representative PRM results from a single patient showing progressive bone density changes in a vertebra harboring a metastatic breast cancer lesion (A) and a vertebra with no detectable lesion (B). PRM_HU_ results show progression as BMD changes over time in (A) compared with the vertebra lacking metastatic disease (B). The relative volumes of PRM_HU+_ (red), PRM_HU−_ (blue), and PRM_HU0_ (green; displayed as 100% − PRM_HU0_ for easy comparison) are graphically displayed (top) along with PRM color overlays on the vertebrae to show the spatial context for the bone changes over time (bottom).

Longitudinal monitoring of bone density changes relative to the first available scan using PRM was performed using ξ = 100 HU to reveal localized changes in BMD and to quantify dynamic changes in vertebral structure caused by metastatic disease (Figure 1). Of the 5 patients included in this study, an average of 10 (±2.4) imaging time points were available that extended to 3.8 (±1.1) years before fracture. For the patient in Figure 2, we analyzed 2 different vertebrae: L2 with metastatic breast cancer (Figure 2A) and T8 without detectable metastatic disease (Figure 2B). The latter served as an intrapatient control. PRM revealed temporal changes in BMD localized around the tumor mass (shown as red voxels) beginning between 12 and 24 months from when CT scans were initiated. As shown by the vertical red line in Figure 2A, the rate of increase in BMD changes rapidly accelerated between 24 and 30 months, after which a PVCF event occurred. An additional PRM analysis was accomplished in vertebra T8 without known metastatic disease using the same set of CT scans from the same patient (Figure 2B). As shown in Figure 2A, changes in PRM-detectable alterations in BMD were relatively stable overall (within 20%) during the same 30-month time period (vertical yellow line). The representative cases demonstrate how PRM_HU_ analysis revealed pathologic progression and provided spatial context for bone changes in a color PRM overlay.

The timing of the analysis and PRM threshold was explored for optimal predictability of fracture and sensitivity to bone density changes by varying δ*t*, *t*_1_, and ξ. Because of the primarily osteoblastic nature of these lesions, PRM_HU+_ was found to have the greatest predictive value (Table 1), with a *t*_1_ and δ*t* of 6 months and a ξ of 100 HU (AUC_ROC_ = 0.918). Attributed to the limited number of datasets analyzed, 2 cutoffs were identified for PRM_HU+_ stratification between fracture and control vertebrae: 1.5% (sensitivity/specificity = 1.00/0.71) or 6.3% (sensitivity/specificity = 0.71/1.00), with ACC = 0.857. PRM_HU−_ was not found to be useful for determining fracture risk in this patient population.

**Table 1.** AUC_ROC_ Values for PRM_HU+_ (ξ = 100 HU)

*t*_1_ (mo)	δ*t* (mo)			
	3	6	9	12
3	0.612	0.653	0.694	0.776
6	0.735	**0.918**	0.755	0.714
9	0.878	0.776	0.796	0.510
12	0.714	0.714	0.510	0.388

**Figure 3.**
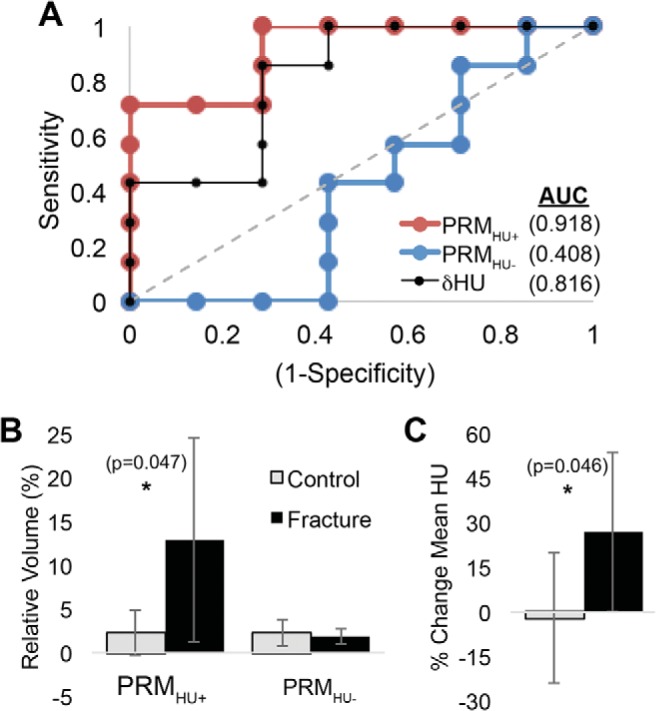
ROC analysis was used to determine optimal imaging time points for PRM detection of PVCFs, which was determined to be *t*_1_ = 6 months before fracture and δ*t* = 6 months. Using these times, ROC curves are displayed for PRM_HU+_ (red), PRM_HU−_ (blue), and the percentage change in mean volume attenuation (δHU; black), with AUC values displayed next to corresponding legend entries (A). Group means were also compared using these time points for PRM values (B) and δHU (C). The asterisk denotes the significant difference between group means found for both PRM_HU+_ and δHU.

In addition to the ROC analysis, group means were also compared (Figure 3C, D). A significant difference was found in PRM_HU+_ (*P* =.047) between PVCF (12.9 ± 11.6%) and controls (2.3 ± 2.5%), but PRM_HU−_ was not significant (*P* =.288). The percentage change in mean bone attenuation (δHU) was also found to be significant (*P* = .046) between PVCF (26.8 ± 26.9%) and controls (−2.2 ± 22.0%).

### Osteolytic Lesions (Case Studies)

**Figure 4.**
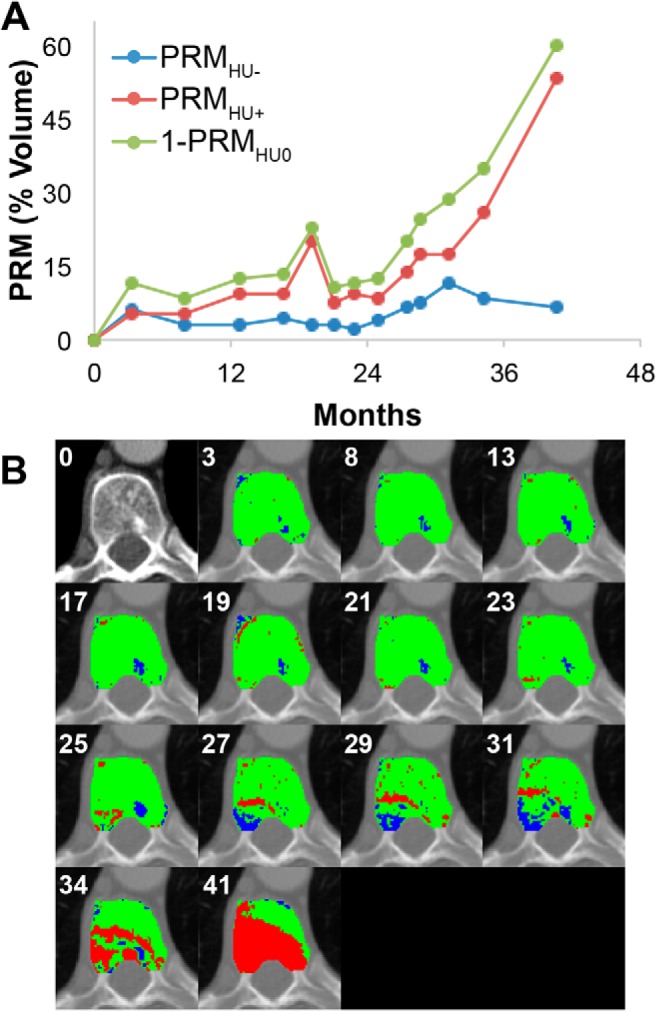
A progressing osteolytic lesion (T9) is tracked by PRM, revealing a moving boundary between the lesion and the surrounding bone visible as a blue (PRM_HU−_) region and a moving red (PRM_HU+_) boundary. Starting at the 12th time point, the lesion appears to shift toward more sclerotic activity (red), leading to bone mineral accumulation.

Two cases of osteolytic lesions were also identified for anecdotal analysis in the thoracic spine, neither of which resulted in fracture within the time limits of the study. These vertebrae served to demonstrate the utility of PRM_HU_ in the presence of osteolytic lesion bone resorption. Because these lesions did not result in PVCFs, they were not included in the previous analysis. In 1 case, shown in Figure 4, the lesion was found to progress during the course of the study. The lesion was visible as a spreading blue volume with a moving red boundary on the interface between the lesion and normal bone. Interestingly, near the end of the imaging time points the lesion seemed to shift in activity from lytic resorption, as evidenced by elevated PRM_HU−_, toward predominantly sclerotic remodeling, as evidenced by a large PRM_HU+_ in the final time point. This sclerotic progression falls in line with the clinical evaluation, with evidence of disease progression in the lumbar spine as demonstrated by (1) worsening lower back pain, (2) a rise in tumor blood markers, (3) magnetic resonance imaging results that showed further lumbar vertebral collapse and increased associated soft tissue, and (4) increased uptake of the L3 and skull lesion via bone scan.

**Figure 5.**
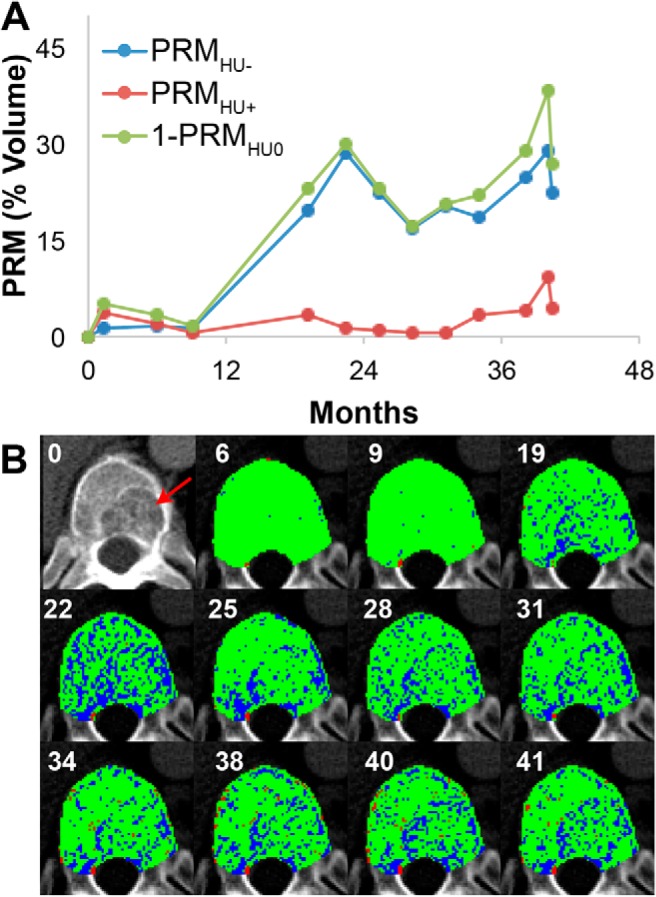
An osteolytic lesion (T11) was visible on the initial CT scan, and subsequent longitudinal PRM analysis shows a substantial reduction in bone density at around 20 months that was visible as a diffuse pattern of PRM_HU−_ (blue).

In another clinical case study, the patient had preexisting bone deterioration before the imaging study; the red arrow in the first image of Figure 5B indicates this deterioration as a region of hypoattenuation in the lower right-hand corner of the vertebral body. Longitudinal analysis of this lesion by PRM_HU_ revealed no focal progression; however, a diffuse loss of bone density throughout the bone was apparent starting at about 19 months (fifth time point), as evidenced by the increased number of disseminated blue voxels. This loss in bone mass may be caused by any combination of (1) osteoporosis, (2) tumor infiltration, (3) fatty infiltration, or (4) hemangioma.

## Discussion

The goal of this study was to apply the PRM methodology to standard CT imaging as a proof of concept for evaluating fracture risk in patients with bone metastases. The availability of regularly acquired longitudinal CT images provided a unique opportunity for the first clinical application of PRM for predicting risk of PVCFs in patients with metastatic breast cancer. Although limited to cases of PVCFs in this study, there are a multitude of bone-involved diseases resulting in SREs, such as osteoporosis. We expect that PRM also will be a robust metric for changes in BMD associated with other diseases.

The current standard for determining fracture risk in patients with bone metastases is BMD measured by dual x-ray absorptiometry. This method has seen widespread clinical use and has shown an impressive predictive value with a relative risk of 2.3 per SD change (22). However, research has indicated that fracture risk reduction in patients treated with bisphosphonates does not correlate well with changes in DXA-derived BMD measurements (23). Global measurements of BMD by DXA are intrinsically insensitive to bone geometry, which is known to greatly affect overall bone strength and may be easily skewed by aortic calcification and other artifacts (24). Investigating the finite element analysis of bone microstructure for determining bone fragility has shown promise (25, 26), but the computational and processing complexity of this method precludes routine clinical use.

The main strengths of PRM as applied to longitudinal CT imaging include (1) sensitivity to localized changes in density that may be masked when using whole-volume statistics, especially at early time points, and (2) resulting classification PRM maps that provide spatial context to these changes. PRM analysis of a rat model of osteoporosis revealed an early drop in bone density that preceded whole-bone measurements, with observable trabecular deterioration as well as cortical expansion for compensation (20). Although PRM does not directly quantify bone strength, small focal changes in mineralization are detectable and may be used to assess relative progression in fracture risk. Furthermore, PRM requires only simple calculations after image coregistration and simple application of proper thresholds for detecting significant change (21). The lesions analyzed in this study were primarily osteoblastic, resulting in bone remodeling, and the accumulation of a mineralized structure, as seen by PRM_HU+_ (red). Although vertebrae with known lesions and resulting fractures generally showed an increasing trend in PRM_HU+_ toward fracture (Figure 2A), control vertebrae (without known lesions during the study time frame) did not present any clear progression in bone density changes (Figure 2B). Because the analyzed lesions presented with primarily osteoblastic activity, the value of PRM_HU+_ was found to be the most predictive of fracture. Although the change in mean vertebral attenuation was also found to be significant between groups in this case (Figure 3D), we believe that larger patient populations would likely include patients with mixed osteolytic/osteoblastic lesions. Such cases would undoubtedly obfuscate whole-volume histogram analysis results with a mixed increase and decrease in bone density, whereas PRM can separately quantify both (illustrated using 2 osteolytic lesions in Figures 4 and 5). Future studies involving lesions with osteoblastic or mixed activities (osteolytic) will require proper analysis to provide for suitable predictive accuracy to be established.

The balance between image resolution and acquisition time (also noise or radiation dose) is a well-known tradeoff. Finite element analysis requires very high resolution and low noise to accurately model the mineralized structure and determine bone strength, which in turn requires greater acquisition time and a significantly higher dose. In vivo or clinical application of this method is therefore not often feasible with current CT technology. The PRM method does not require such high resolution and thus, as shown in this study, can provide sensitive results on relatively low-resolution images. Moreover, through simultaneous quantification and spatial display of both increasing and decreasing bone density, an indication of the degree of osteolytic/osteoblastic activity of the lesion may be ascertained. In addition to risk assessment, the spatial maps of density change may prove useful for planning orthopedic strategies if necessary.

Further studies are necessary to fully explore the quantification of fracture risk progression using this technique, which would ideally monitor all lumbar and thoracic vertebrae simultaneously. This would necessitate an automated segmentation routine for efficient processing and clinical accessibility and scalability. In addition, the acquired image resolution as well as reconstructed image noise may substantially affect detected density changes as a result of the partial-volume effect and limited ability of the coregistration process to accurately interpolate bone tissue interfaces. Although a comprehensive assessment is beyond the scope of this preliminary study, the results presented are very promising and present PRM as a potentially novel image biomarker for early detection of skeletal-related events in cancer patients.

## Summary

The overall purpose for accurately predicting vertebral fracture risk is to provide clinicians with the required evidence for undertaking intervention. Bone density analysis through PRM provided a significant indication of an impending fracture with an imaging frequency of twice per year—ample time for adjusting patient care and possibly improving patient quality of life when applied prospectively. Spatial maps of density changes revealed focal bone remodeling and could provide much-needed context for corrective intervention and potentially a greater sensitivity to fracture-related bone changes than whole-bone analysis, especially in the case of mixed osteolytic/osteoblastic lesions. Ultimately, PRM is a sensitive and flexible image biomarker that can be developed for clinical use as an indicator of disease and fracture risk progression.
